# Family based screening and smoking cessation for COPD detection: Hybrid cross-sectional and single-arm, multicenter intervention study

**DOI:** 10.18332/tid/235829

**Published:** 2026-10-22

**Authors:** Carlos Rábade-Castedo, Carlota Rodríguez-García, Paula Lado-Argibay, Goretti Fernández-Morón, Patricia Susana Vázquez-Millán, Juana Antonia Fernández-Moreno, Tara Pereiro-Brea, Sandra Buján-Garmendia, María Cruz Carbajales-Bouzón, María José Breijo-Pena, Elivia Canosa-Santos, Tamara Varela-Sanda, Elena Fernández-Fernández, Laura Vela-Flórez, José Rubén Blanco-Rodríguez, Marta Dablanca-Pallares, Pablo Mascareñas-Pazos, María Elena Toubes-Navarro, Carlos Zamarrón-Sanz, Luis Valdés-Cuadrado

**Affiliations:** 1 Department of PulmonologyComplejo Hospitalario Universitario de Santiago de CompostelaSantiago de CompostelaSpain; 2 Health Research Institute of Santiago de Compostela (IDIS)Santiago de CompostelaSpain; 3 Department of PulmonologyHospital Arquitecto MarcideFerrolSpain; 4 Family and Community Medicine, Melide Primary Care CenterMelideSpain; 5 Family and Community Medicine, Noia Primary Care CenterSantiago de CompostelaSpain; 6 Family and Community Medicine, Bembibre-Val do Dubra Primary Care CenterSantiago de CompostelaSpain; 7 Family and Community Medicine, Ordes Primary Care CenterSantiago de CompostelaSpain; 8 Family and Community Medicine. Santa Comba Primary Care CenterSantiago de CompostelaSpain; 9 Department of PulmonologyComplejo Hospitalario Universitario de Santiago de Compostela, Nursing, Case Management, COPD UnitSantiago de CompostelaSpain; 10 Department of PulmonologyComplejo Hospitalario Universitario de Santiago de Compostela, Nursing, Respiratory Functional Exploration UnitSantiago de CompostelaSpain; 11 Department of PulmonologyComplejo Hospitalario Universitario de Santiago de Compostela, Nursing, Smoking Cessation UnitSantiago de CompostelaSpain; 12 Family and Community Medicine, Vila de Cruces Primary Care CenterSantiago de CompostelaSpain; 13 Family and Community Medicine, Rianxo Primary Care CenterSantiago de CompostelaSpain; 14 Family and Community Medicine, Mazaricos Primary Care CenterSantiago de CompostelaSpain; 15 Family and Community Medicine, Lousame Primary Care CenterSantiago de CompostelaSpain; 16 Complejo Hospitalario Universitario de Santiago de Compostela, Nursing, Emergency DepartmentSantiago de CompostelaSpain; 17 School of Nursing, University of Santiago de CompostelaSantiago de CompostelaSpain; 18 Medicine School, University of Santiago de CompostelaSantiago de CompostelaSpain

**Keywords:** diagnosis, smoking cessation, treatment, COPD, family environment

## Abstract

**Introduction:**

Chronic obstructive pulmonary disease (COPD) is the third leading cause of morbidity and mortality, making early detection crucial to improving prognosis. This study aimed to identify new cases of COPD, clinical profiles, and predictors among smokers and their cohabiting family members seeking help to quit smoking.

**Methods:**

A hybrid cross-sectional and single-arm intervention study was conducted over one year across 8 Primary care centers in the Santiago-Barbanza health area (Galicia, Spain), including 217 participants (index smokers and cohabitants without contraindications for spirometry). Clinical variables for diagnosis and characterization of the patient with COPD were analyzed, as well as variables associated with smoking (degree of smoking, motivation, nicotine dependence or abstinence at 6 months). The intervention involved: 1) performing forced spirometry on active smokers and cohabitants exposed to tobacco smoke for >20 years; and 2) enrolling smoking family members into a cessation program. Descriptive statistics, univariate analysis, and multivariate logistic regression were used to identify independent risk factors (adjusted odds ratio, AOR, and 95% CI).

**Results:**

Out of 217 participants (104 family members), 147 (67.8%) were active smokers. Over 40% reported respiratory symptoms, with dyspnea being the most frequent. Spirometric abnormalities were present in 99 participants (45.6%), identifying 53 new COPD cases (24.4%), with 58.5% in the mild stage (GOLD I). Among cohabitants, 17 additional cases (15%) were detected regardless of smoking status. Cumulative smoking exposure time (AOR=1.04; 95% CI: 1.03–1.10; p=0.006) and respiratory symptoms (AOR=3.90; 95% CI: 1.25–10.00; p=0.036) were independent risk factors. This strategy led to the inclusion of 38 additional smokers in the cessation program, 25 of whom successfully quit.

**Conclusions:**

Active screening for COPD in smokers and their cohabitants facilitates early-stage diagnosis, potentially altering disease progression while increasing family engagement in smoking cessation programs.

## Introduction

Chronic obstructive pulmonary disease (COPD) is one of the most prevalent diseases in our environment, representing the third leading cause of morbidity and mortality worldwide^1^. Exposure to tobacco or other harmful agents, the presence of respiratory symptoms such as dyspnea, cough, and sputum production, and chronic airflow limitation, documented by post-bronchodilator spirometry, are the three criteria necessary for the diagnosis of this disease^2^. Despite its high prevalence and the availability of well-established diagnostic criteria, COPD remains a widely underdiagnosed disease^3^, especially in its early stages when interventions can most effectively modify its clinical course and prognosis^3-7^.

Current COPD screening strategies have focused on smokers, using clinical questionnaires, portable devices, or opportunistic spirometry in at-risk populations. Although these approaches have proven useful, their diagnostic performance is variable and they do not systematically address a key setting of prolonged tobacco exposure: the smoker’s family unit^8-17^.

The family setting of a smoker represents a context of particular interest from a clinical and preventive perspective. Those living with the smoker share continuous exposure to tobacco smoke for years, in many cases from early stages of life, and can accumulate significant respiratory damage even in the absence of active smoking. Furthermore, living with a smoker is associated with a higher probability of active smoking, greater difficulty in quitting, and a higher risk of chronic respiratory diseases. However, actively screening for COPD in those living with a smoker is not part of routine clinical practice^18^.

Smoking cessation clinics in primary care offer a unique opportunity to identify not only at-risk smokers but also their cohabitants, allowing for a systematic assessment of lung function in a population group with high susceptibility and which is usually under-evaluated^16-19^. This family-centered approach could significantly contribute to reducing the underdiagnosis of COPD, detecting the disease in its early stages, and recognizing emerging profiles such as pre-COPD or COPD associated exclusively with passive exposure to tobacco smoke^18-20^.

Therefore, the objective of this study is to determine if it is feasible to diagnose new cases of COPD using a household member screening.

## Methods

### Design

A hybrid cross-sectional and single-arm, multicenter intervention study was conducted with a recruitment period between September 2023 and September 2024, involving patients from the same health area. This study complies with the Equator Network’s STROBE guidelines for observational studies (Supplementary file Table S1)

### Population

Smokers attending primary care clinics for any reason, as well as their cohabitants, were included. Participants came from eight health centers in the Santiago-Barbanza health area (Arca, Bembibre, Boimorto, Lousame, Ordes, Rianxo, Santa Comba, and Vila de Cruces), Spain, with a population of 40594 inhabitants.

Recruitment began through active smokers (index smokers, those who attended a smoking cessation unit to quit smoking) who attended Primary care, with the subsequent inclusion of their cohabitants.

The following inclusion criteria were considered: 1) being an active smoker or a household member who had resided in the same home as the smoker for at least the last 20 years, with exposure to tobacco smoke exceeding 10 hours per week; 2) agree to participate in the study by signing the informed consent form; and 3) be able to perform forced spirometry and complete the COPD-PS questionnaires (Supplementary file Table S2)^8^ and the study-specific questionnaire (Supplementary file Table S3).

Index smokers or cohabitants were excluded if they refused to participate, were unable to complete questionnaires or perform forced spirometry due to physical or mental limitations, lacked signed informed consent, or presented absolute contraindications for spirometry

A sample size of 202 subjects (95% CI, 3% precision) was estimated, expanded to 238 after considering a 15% loss.

### Intervention

The intervention was carried out in two phases: Primary care (Phase 1) and Specialized care (Phase 2) (Figure 1).

**Figure 1:**
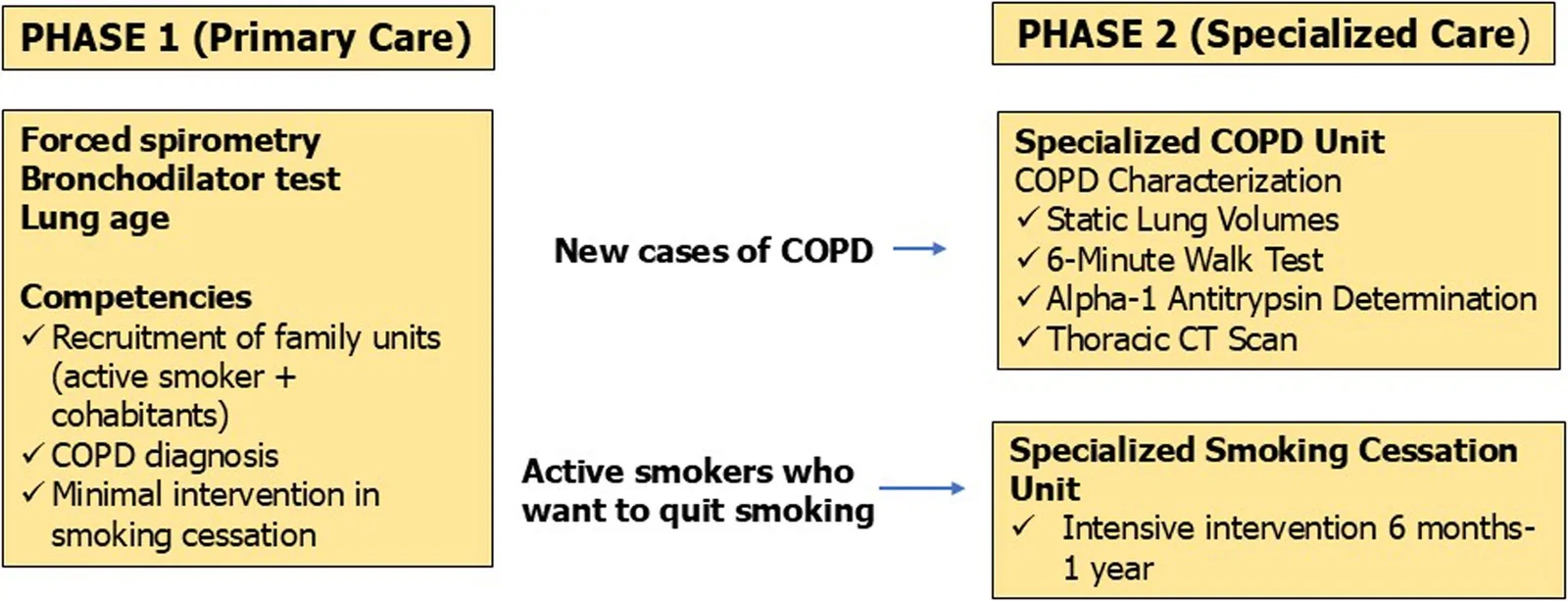
Two-phase family-based intervention workflow for COPD detection and smoking cessation across primary and specialized care settings, Santiago-Barbanza, Spain, September 2023–September 2024 (N=217)

#### Phase 1: Primary care

Recruitment of family units consisting of the index smoker (index case) and their cohabitants was carried out after explaining the project and obtaining informed consent. In this phase, the COPD-PS questionnaire^8^ and another questionnaire assessing passive exposure to tobacco smoke were completed (Supplementary file Tables S2 and S3), in addition to baseline and post-bronchodilation spirometry using a Care Fusion Masterscreen APS Series 06xo222 spirometer.

Smokers were offered brief health advice and a proposed treatment plan for smoking cessation. Recommendations were also provided to reduce exposure to secondhand smoke in the home environment.

#### Phase 2: Specialized care

Two groups of participants were included in specialized care. Participants diagnosed with COPD, defined by the presence of respiratory symptoms associated with a post-bronchodilator obstructive pattern, were referred to the specialized COPD unit for disease characterization (Figure 1). Prepared smokers who were unable to quit with the minimal intervention and treatment offered by primary care professionals were referred to a specialized smoking cessation unit.

The smoking cessation intervention carried out in the specialized smoking cessation unit provided an individualized, multidisciplinary approach led by medical and nursing professionals. Formally structured across seven consultations – an initial baseline visit and six follow-up sessions – the intervention began with psychological support via cognitive-behavioral therapy alongside tailored pharmacological treatment (nicotine replacement therapy, bupropion, or cytisinicline). In subsequent visits, abstinence was systematically verified, accomplishments were reinforced, and practical strategies were provided to overcome resistance and manage cravings. Additionally, patients received self-help materials and ongoing follow-up for 6 to 12 months.

### Study variables

The study variables were categorized according to their methodological role. Demographic variables included sex (male, female), age, education level (primary school, secondary school, university), and occupation.

Regarding smoking-related variables, active and passive tobacco exposure were evaluated through smoking status (active smoker, passive smoker, non-smoker), cumulative tobacco consumption (pack-years), cohabitation with an index smoker (yes, no), length of cohabitation, and both the duration and intensity of smoke exposure. To objectively confirm smoking status, all participants underwent exhaled carbon monoxide CO-oximetry (ppm) and a qualitative urine cotinine test (positive, negative).

Spirometric variables were evaluated as continuous parameters before and after bronchodilator testing using standard maneuvers: forced expiratory volume in 1 seconds (FEV1, expressed in L and % predicted value), forced vital capacity (FVC, in L and % predicted value), the FEV1/FVC ratio (%), and forced expiratory flow between 25% and 75% of FVC (FEF 25–75%, in L/s and % predicted value).

Diagnostic outcome variables were analyzed as categorical dichotomous variables (presence, absence) for both smokers and cohabitants, encompassing: 1) pathological spirometry, 2) obstructive ventilatory impairment, 3) new diagnosis of COPD, and 4) presence of other respiratory diseases.

In the subgroup of participants with a newly diagnosed COPD, clinical severity and disease characterization were assessed using: degree of dyspnea (modified Medical Research Council [mMRC] scale, range 0–4)^21^, health-related quality of life (COPD Assessment Test [CAT] questionnaire, range 0–40)^21^, level of physical activity, number of exacerbations and hospitalizations in the last year, peripheral blood eosinophil count (cells/mL, BODEX index score (range 0–10)^22,23^, diffusing capacity of the lung for carbon monoxide (DLCO, % predicted value), static lung volumes (% predicted value) and chest X-ray pattern.

Finally, in the subgroup of active smokers, the following variables were analyzed: cumulative tobacco consumption (pack-years), stage of change (Prochaska and DiClemente model)^24^, levels of motivation to quit and self-efficacy (evaluated using a visual analog scale [VAS], range 0–10), nicotine dependence (Fagerström test for nicotine dependence [FTND], range 0–10)^25^, number and proportion of smokers who made an attempt to quit smoking with this strategy (yes, no), and confirmed smoking abstinence at 6 months post-intervention (yes, no). The continuous abstinence rate, defined as the absolute absence of any tobacco use from the day the decision to quit was made until 6 months and verified by CO-oximetry (CO <10 ppm and negative cotinine test).

### Ethical aspects

The study was approved by the Clinical Research Committee (CEIC) of Santiago de Compostela - Lugo (code 2022/295).

### Statistical analysis

Data are presented as mean ± standard deviation (SD) or median with interquartile range (IQR), as appropriate. Descriptive statistics were used to summarize patient characteristics.

Comparisons between groups (participants with vs without COPD) were performed using Student’s t-test for normally distributed continuous variables or the Mann–Whitney U test for non-parametric continuous variables. Categorical variables were compared using the chi-squared test or Fisher’s exact test, as appropriate.

Multivariate analysis was performed using binary logistic regression. The variables used in the multivariate regression analysis were selected *a priori* based on their clinical relevance, conceptual plausibility, and the results of the univariate analyses. The results are presented as adjusted odds ratios (AORs) with 95% confidence intervals (CIs).

All statistical tests were two-sided, and p<0.05 were considered statistically significant. Analyses were performed using IBM SPSS Statistics version 20.0 (IBM Corp., Armonk, NY, USA)^26^.

## Results

### Descriptive characteristics of the sample

A total of 257 participants (135 index smokers and 122 cohabitants) were initially recruited from eight health centers in the Santiago de Compostela-Barbanza area (Table 1). Of these, 40 individuals were sequentially excluded: 3 cohabitants declined signed informed consent, 29 index smokers were excluded due to the lack of participating household cohabitants, 4 cohabitants had <20 years of passive smoke exposure, and 4 participants (2 index smokers and 2 cohabitants) were unable or declined to perform forced spirometry. Consequently, the final analyzed sample consisted of 217 participants (104 index smokers and 113 cohabitants) (Figures 2 and 3).

**Table 1:** Baseline demographic and clinical characteristics of participants in a family-based COPD screening study in primary care, Santiago-Barbanza, Spain, September 2023–September 2024 (N=217)

Characteristics	Index smokers ^a^ (N=104) n (%)	Cohabitants (N=113) n (%)	Total (N=217) n (%)
**Age** (years), mean ± SD	54.3 ± 10.1	51.8 ± 14.5	53.0 ± 12.6
**Sex**			
Male	70 (67.3)	33 (29.2)	103 (47.5)
Female	34 (32.7)	80 (70.8)	114 (52.5)
**Smoking status**			
Active smoker	103 (99.0)	44 (38.9)	147 (67.7)
Non-smoker/ex-smoker	1 (1.0)	69 (61.1)	70 (32.3)
**History of active smoking**			
Yes	104 (100.0)	60 (53.1)	164 (75.6)
No	0 (0.0)	53 (46.9)	53 (24.4)
**Spirometric abnormality**			
Present	64 (61.5)	35 (31.0)	99 (45.6)
Absent	40 (38.5)	78 (69.0)	118 (54.4)
**Respiratory symptoms**			
Present	52 (50.0)	35 (31.0)	87 (40.1)
Absent	52 (50.0)	78 (69.0)	130 (59.9)
**New COPD diagnosis**			
Yes	36 (34.6)	17 (15.0)	53 (24.4)
No	68 (65.4)	96 (85.0)	164 (75.6)

a Index smokers: those who attended a smoking cessation unit to quit smoking.

**Figure 2:**
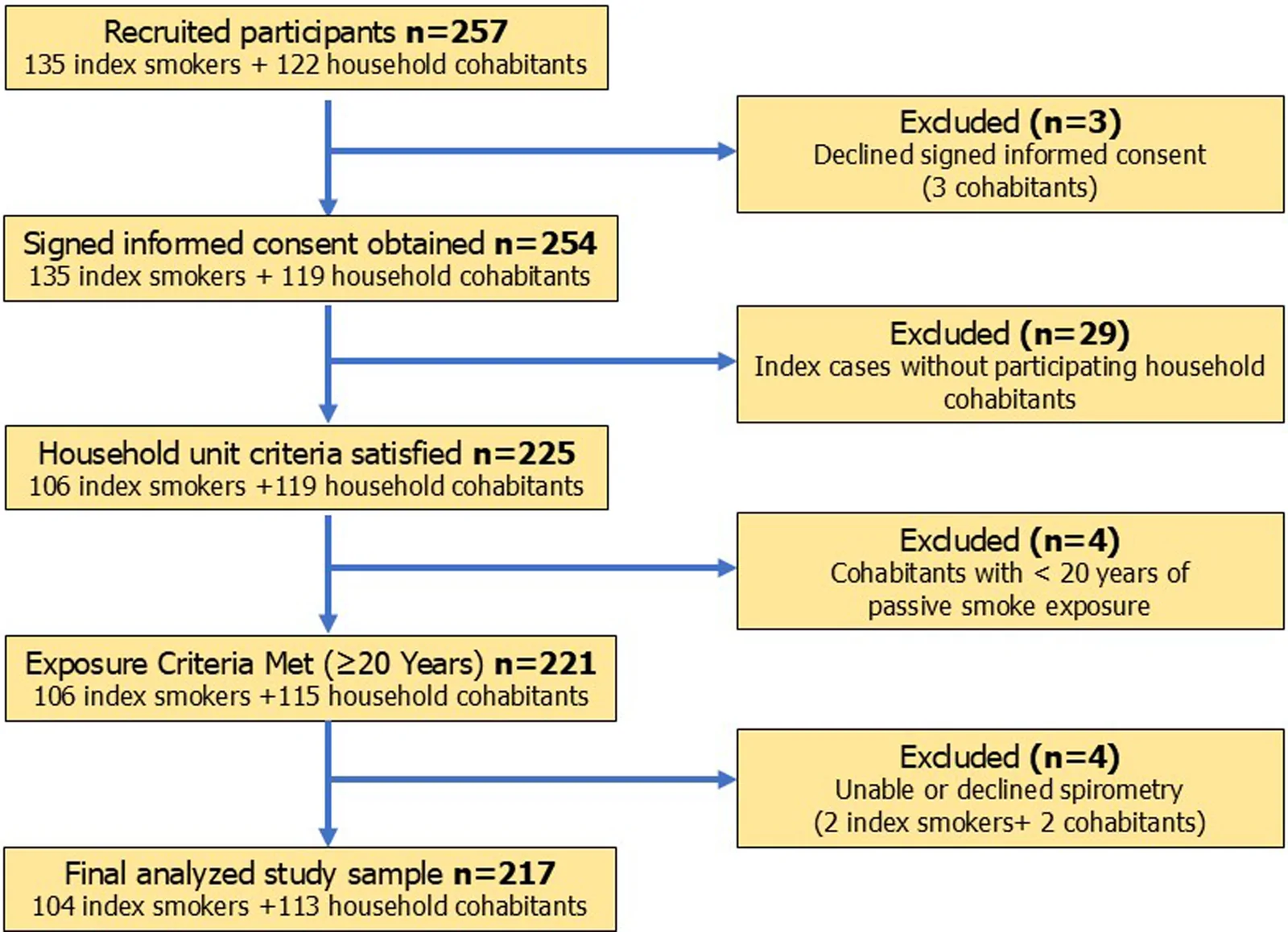
Recruitment and eligibility screening flowchart of participants in a primary care COPD screening study, Santiago-Barbanza, Spain, September 2023–September 2024 (N=257 recruited, N=217 analyzed)

**Figure 3:**
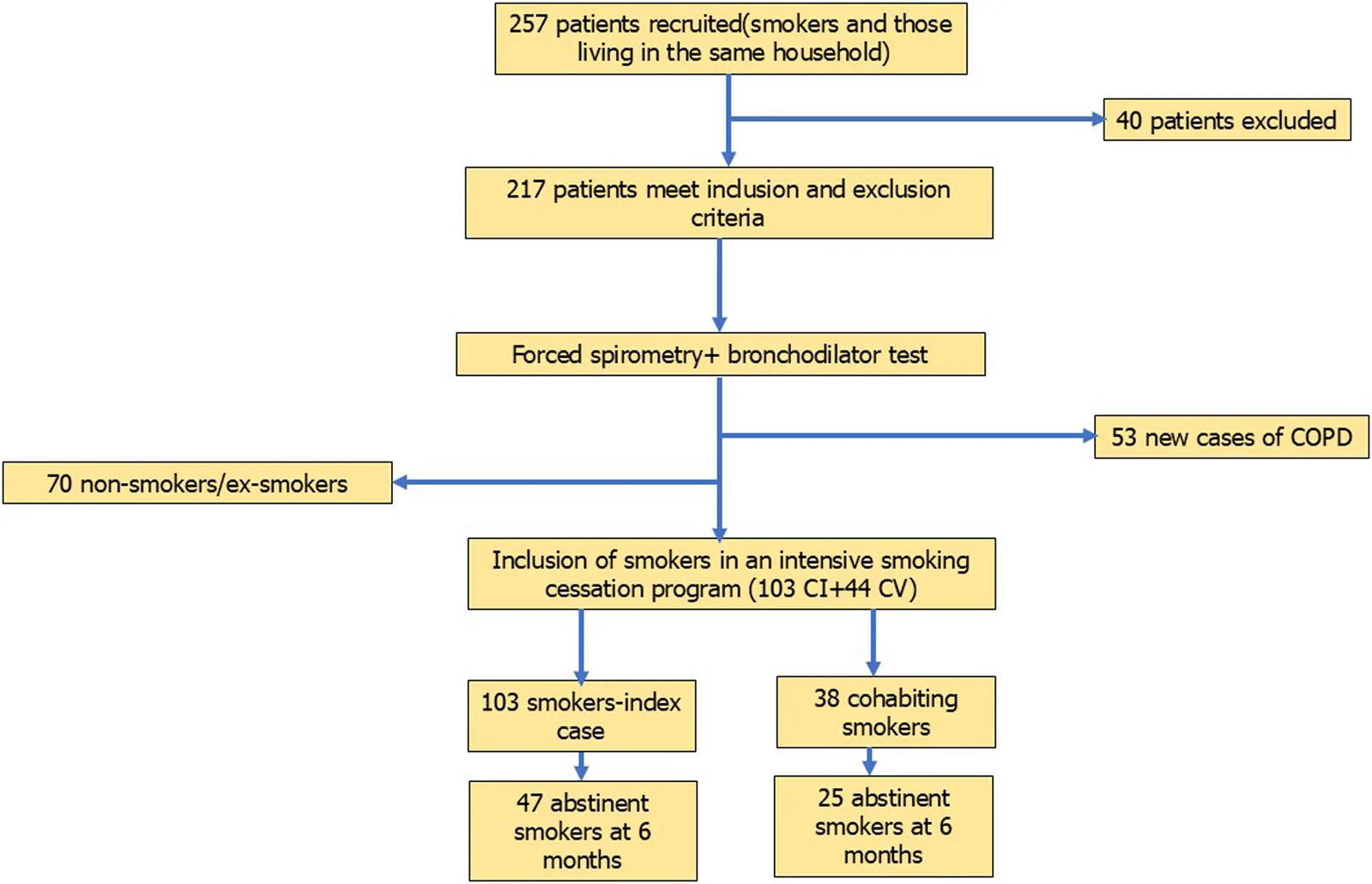
Flowchart of participant progression through spirometry screening, COPD diagnosis, and 6-month smoking cessation intervention outcomes, Santiago-Barbanza, Spain, September 2023–September 2024 (N=217)

Regarding the two-phase intervention workflow (Figure 1), during Phase 1 (Primary care), all 217 participants underwent baseline evaluation, completing the COPD-PS^8^ and passive smoke exposure questionnaires alongside spirometry with bronchodilator testing. All active smokers received brief cessation advice. Following Phase 1 screening, participants were navigated to Phase 2 (Specialized care) based on diagnostic and clinical criteria (Primary care COPD diagnosis or the need for intensive smoking cessation intervention).

Overall, 104 families were studied. The mean family size was 2.02 members per family. The sample consisted of 103 men (47.5%) and 114 women (52.5%), with a mean age of 53.0 ± 12.6 years (range: 20–85 years).

Of the 217 participants, 147 (67.7%) were current smokers and 70 (32.3%) were non-smokers or former smokers. A total of 164 participants (75.6%) had a history of smoking. The mean duration of passive exposure to tobacco smoke was 18.5 ± 15.3 years, while the mean duration of active exposure was 33.8 ± 11.9 years, with a total cumulative exposure of 49.8 ±17.4 years.

A total of 40.1% of participants reported respiratory symptoms, with dyspnea being the most frequent symptom (62 participants; 71.3% of cases), followed by morning cough (50; 57.4%), expectoration (38; 43.6%), and recurrent respiratory infections (16; 18%). Of the 62 participants who presented with dyspnea, 51 (82.3%) reported experiencing it ‘sometimes’, 9 (14.5%) ‘most of the time’, and 2 (3.2%) ‘all the time’. Of the patients who presented with cough and/or expectoration, 76% reported experiencing it ‘a few days a month’, and the rest (24%) ‘almost every day of the week’. Of the 87 participants with respiratory symptoms, 72 patients (83%) were unaware that they had to reduce their physical activity as a consequence of these symptoms.

Smokers showed a significantly higher prevalence of respiratory symptoms compared to non-smokers exposed only to secondhand smoke (51.2% vs 28.6%; OR=2.61; 95% CI: 1.39–4.90; p=0.005).

All participants underwent forced spirometry with a bronchodilator test, meeting quality and reproducibility criteria. The mean FEV1 and forced mid-expiratory flow (FEF25-75%) values were 98.3 ± 17.4% and 90.4 ± 3 5.0% of predicted, respectively.

Mean FEV1 was significantly lower in active smokers than in those exposed to secondhand smoke (93.8 ± 17.9% vs 106.5 ± 15.1%; p<0.05).

A total of 99 participants (45.6%) presented spirometric abnormalities. Of these, 54 (54.1%) showed obstructive ventilatory impairment defined by an FEV1/FVC ratio <0.7 post-bronchodilator. Another 25 participants (25.5%) presented a non-obstructive ventilatory pattern with bronchial reversibility criteria and 18 participants (18.4%) showed a non-obstructive pattern with impaired mid-expiratory flows (FEF25–55 <60%). In one case (1%), a pattern compatible with non-obstructive ventilatory impairment was observed.

Among patients with spirometric abnormalities 83 (84%) were active smokers, 9 (9%) were former smokers, and 7 (7%) were non-smokers.

Fifty-three new cases of COPD were diagnosed, representing 24.4% of the analyzed sample, defined by the presence of respiratory symptoms, a post-bronchodilator obstructive pattern, and the exclusion of other causes.

According to the Spanish COPD Guideline (GesEPOC 2025) classification^2^, 33 patients (62.23%) had low-risk COPD, 10 (18.89%) had high-risk non-exacerbator COPD, 7 (13.21%) had high-risk eosinophilic exacerbator COPD, and 3 (5.67%) had high-risk non-eosinophilic exacerbator COPD.

Active smoking was the predominant condition across all COPD phenotypes.

In the low-risk group 28 (84.84%) were active smokers, 4 (12.12%) never smokers (exposed exclusively to secondhand smoke), and 1 (3%) former smoker. In both the high-risk non-exacerbator and high-risk eosinophilic exacerbator groups, all patients were active smokers (100%). Finally, among high-risk non-eosinophilic exacerbator patients, 2 (66.7%) were active smokers and 1 (33.3%) was a former smoker (Supplementary file Table S4).

When categorized by spirometric severity, according to the Global strategy for diagnosis, management, and prevention of COPD (GOLD) classification^21^, 31 patients (58.5%) were stage I, 15 (28.3%) stage II, 4 (7.5%) stage III, and 3 (5.7%) stage IV. Regarding the GOLD groups, 27 patients were classified as group A, 16 as group B, and 10 as group E. Active smoking predominated across all groups: GOLD A (22; 81.5% active smokers), (4; 14.8% never smokers), (1; 3.7% former smokers), GOLD B (16; 100% active smokers) and GOLD E [9 (90%) active smokers and 1 (10%) former smoker) (Supplementary file Table S4).

In addition, early respiratory conditions and other non-COPD pathologies were identified: 24 patients (11%) met pre-COPD criteria^27^. Of these, 71.4% had a history of smoking. Seven patients (3.1%) were diagnosed with PRISm (Preserved Ratio Impaired Spirometry), all with a history of smoking^28^. Finally, 10 patients (4.4%) were diagnosed with asthma according to the criteria from the Asthma Guideline (GEMA 5.4)^29^, and 4 patients (1.8%) presented with other respiratory pathologies (diffuse interstitial lung disease, bronchiectasis, and obstructive sleep apnea).

### Analysis of cohabitants

In total, 113 family members living with index smokers were evaluated from the 104 families included in the study. Of these, 33 (29.2%) were male, with a mean age of 51.8 ± 14.5 years. The cohabitation relationship was predominantly with a partner (85 cases; 75.2%), followed by children (13; 11.5%), siblings (8; 7%), and parents (7; 6.2%).

Regarding tobacco use, 44 cohabitants (38.9%) were current smokers, 52 (46%) had never smoked, and 17 (15%) were former smokers. Overall, 52.7% of the evaluated cohabitants reported a history of smoking.

Respiratory symptoms were present in 31% of cohabitants. Among the 35 cohabitants with detected spirometric abnormalities, 14 were non-active smokers and 7 had exclusively passive exposure to tobacco smoke.

COPD was diagnosed in 17 cohabitants (15%). Of these, 11 (64.7%) were active smokers, while 13 (76.9%) had a history of smoking. Four cohabitants (23.5%) diagnosed with COPD had never smoked, with passive exposure to tobacco smoke identified as the sole etiological factor. Combined exposure to active and passive tobacco smoke was observed in 10 cohabitants (61.5%) with COPD.

Likewise, 13 cohabitants met pre-COPD criteria^27^, of whom 8 had a history of smoking and 5 had never smoked. Five cohabitants were diagnosed with PRISm^28^, all of them with a history of smoking. Finally, 6 cohabitants (5.3%) were diagnosed with asthma according to the GEMA 5.4 guidelines^29^. Of these, 5 were not active smokers and 1 was a smoker, with 80% of cases identified as having exclusive exposure to secondhand smoke.

### Analysis of active smokers

A total of 147 active smokers were identified. Of these, 103 (70%) were index smokers, meaning those who attended the smoking cessation unit to quit smoking. The mean cumulative tobacco consumption was 38.9 ± 15.4 pack-years, with a mean nicotine dependence score of 5.9 ± 1.1. The mean motivation level measured through the visual analog scale was 7.8 ± 2.1. An additional 29.9% (44/147) were cohabitants and, therefore, additional smokers identified through this approach.

The continuous abstinence rate, defined as the absolute absence of any tobacco use from the day the decision to quit was made until 6 months and verified by CO-oximetry (CO <10 ppm), was 49% (72/147). All index smokers made a structured quit attempt, with 45.6% (47/103) maintaining abstinence at 6 months.

Among cohabiting smokers, 38/44 (86.4%) participated in an intensive smoking cessation intervention, with 25 smokers (56.8%; 25/44) successfully quitting, and 65.8% (25/38) of those who made a quit attempt. Overall, this strategy enabled 38 smokers to further attempt to quit through an intensive intervention, and 25 of them successfully quit smoking. In other words, by expanding the number of smokers targeted for intervention (cohabiting smokers), the number of those receiving smoking cessation treatment increased by 22.8%, and the number who quit smoking increased by 17%.

Participants with spirometric impairment showed a slightly higher continuous abstinence rate at 6 months compared to those with normal lung function (52% vs 47.9%), although this difference did not reach statistical significance (OR=1.18; 95% CI: 0.69–2.01; p=0.498). Similarly, when analyzing outcomes stratified by diagnostic status, smokers diagnosed with COPD demonstrated a higher cessation rate compared to those without COPD (55.6% vs 46.4%), though this difference was likewise not statistically significant (OR=1.44; 95% CI: 0.70–2.99; p>0.05).

### Factors associated with developing COPD in the sample

Univariate analysis showed that the frequency of patients with COPD was significantly higher in current smokers (47 patients, 88.8%) compared to non-smokers (6 patients; 11.3%; p=0.001), as well as in those with a history of smoking (49 patients; 92.5%) compared to those without such a history (4 patients; 7.5%; p=0.000). Active smokers in the sample presented significantly lower mean FEV1 values (94.8 ± 12% vs 105.5 ± 12%; p=0.000) and FEF25–75 (83.7 ± 34 vs 104.4 ± 32; p=0.000). A higher frequency of developing COPD was observed in older patients in the sample (61.3 ± 7.8 vs 50.6 ± 12.7; p=0.001), those with higher cumulative cigarette consumption in pack-years (50.8 ± 9.7 vs 32.3 ± 17.2; p=0.001), and those with a greater number of years of active smoking (36.9 ± 14.8 vs 23.3 ± 15.9; p=0.000).

Of the COPD cases, 11.3% were identified in participants with exclusive passive exposure to tobacco smoke. Among non-smokers with passive exposure, individuals diagnosed with COPD had significantly more years of passive smoke exposure compared to those without COPD (39.7 ± 9.8 vs 27.5 ± 10.1 years; p=0.027) (Table 2).

**Table 2:** Univariate analysis comparing demographic, clinical, and tobacco exposure characteristics between participants with and without COPD in a primary care family-based screening study, Santiago-Barbanza, Spain, September 2023–September 2024 (N=217)

Variables	COPD (N=53) n (%)	No COPD (N=164) n (%)	Total (N=217) n (%)	p
**Age** (years), mean ± SD	61.3 ± 7.8	50.6 ± 12.7	53.0 ± 12.6	0.001
**Sex**				0.000
Male	31 (58.5)	59 (36.0)	90 (41.5)	
Female	22 (41.5)	105 (64.0)	127 (58.5)	
**Education level**				0.890
Basic or no education	17 (53.1)	43 (48.3)	60 (49.6)	
Secondary or higher	15 (46.9)	46 (51.7)	61 (50.4)	
**Participant role**				0.000
Index case	36 (67.9)	68 (41.5)	104 (47.9)	
Cohabitant	17 (32.1)	96 (58.5)	113 (52.1)	
**Smoking status**				0.000
Active smoker	47 (88.7)	100 (61.0)	147 (67.7)	
Non-smoker/ex-smoker	6 (11.3)	64 (39.0)	70 (32.3)	
**History of active smoking**				0.000
Yes	49 (92.5)	115 (70.1)	164 (75.6)	
No	4 (7.5)	49 (29.9)	53 (24.4)	
**Exclusive passive tobacco exposure**				0.073
Yes	6 (11.3)	83 (50.6)	89 (41.0)	
No	47 (88.7)	81 (49.4)	128 (59.0)	
**Duration of exclusively passive smoking** (years), mean ± SD	39.7 ± 9.8	27.5 ± 10.1	28.7 ± 10.6	0.027
**Duration of active smoking** (years), mean ± SD	36.9 ± 14.8	23.3 ± 15.9	26.8 ± 16.9	0.000
**Cumulative tobacco exposure** (pack-years), mean ± SD	50.8 ± 9.7	32.3 ± 17.2	35.6 ± 17.6	0.001
**Respiratory symptoms**				0.000
Present	42 (79.2)	45 (27.4)	87 (40.1)	
Absent	11 (20.8)	119 (72.6)	130 (59.9)	
**FEV1** (% predicted), mean ± SD	83.8 ± 20.8	102.6 ± 13.5	98.3 ± 17.4	0.000
**FEF 25–75%** (% predicted), mean ± SD	47.8 ± 15.5	102.8 ± 28.6	90.4 ± 35.1	0.000
**Exhaled CO level** (ppm), mean ± SD	14.4 ± 8.3	12.3 ± 8.9	13.4 ± 8.7	0.470
**Motivation to quit** (VAS score, 0–10), mean ± SD	7.3 ±1.6	7.95 ± 2.2	7.8 ± 2.1	0.310
**Nicotine dependence** (FTND score, 0–10), mean ± SD	6.0 ± 0.8	5.9 ± 1.2	6.0 ± 1.2	0.820
**Smoking abstinence** at 6 months				0.360
Yes	20 (42.6)	52 (52.0)	72 (49.0)	
No	27 (57.4)	48 (48.0)	75 (51.0)	

FEV1: forced expiratory volume in 1 s. FEF 25–75%: forced expiratory flow between 25% and 75% of vital capacity. CO: carbon monoxide. ppm: parts per million. VAS: Visual Analog Scale. FTND: Fagerström test for nicotine dependence.

In the multivariate analysis, we observed two predictive factors for developing COPD: cumulative smoking exposure time, measured in tobacco burden (pack-years, and years of exposure to active and passive smoking) (AOR=1.04; 95% CI: 1.03–1.10; p=0.006), and the presence of respiratory symptoms in the subjects of the sample (AOR=3.90; 95% CI: 1.25–10.00; p=0.036) (Table 3).

**Table 3:** Multivariate logistic regression analysis of independent factors associated with COPD diagnosis among participants in a family-based screening study, Santiago-Barbanza, Spain, September 2023–September 2024 (N=217)

Variables	Reference/measurement scale	AOR	95% CI	p
**Sex**	Female (vs Male)	1.72	0.56–5.20	0.335
**Tobacco exposure duration**	Per 1-year increase (years)	1.04	1.03–1.10	0.006
**Respiratory symptoms**	Present (vs Absent)	3.90	1.25–10.00	0.036
**Nicotine dependence score**	Per 1-point increase in FTND score (0–10)	0.96	0.80–1.14	0.650
**Six-month smoking abstinence**	Achieved (vs Not achieved)	0.50	0.18–1.30	0.17

AOR: adjusted odds ratio. CI: confidence interval. FTND: Fagerström test for nicotine dependence.

## Discussion

In this prospective, multicenter, hybrid intervention study, active case finding for COPD through the screening of active smokers and their long-term cohabitants in primary care made it possible to identify new patients with this disease, predominantly at early stages, and successfully recruit a substantial number of cohabiting smokers into an intensive smoking cessation program. Specifically, 53 new cases of COPD were diagnosed (24.4% of the sample), of whom 17 (15%) were cohabitants, including both active smokers and never smokers with prolonged passive exposure. In addition, cumulative tobacco exposure and the presence of respiratory symptoms were identified as two factors associated with the development of this disease.

Likewise, expanding the smoking cessation intervention to the family setting allowed 38 cohabiting smokers to receive specialized and intensive smoking cessation treatment provided by qualified and accredited personnel.

Overall, these results suggest that Primary care may constitute a suitable setting for integrating active COPD case finding, spirometry, and smoking cessation intervention. This strategy, based on identifying COPD cases through the assessment of lung function in cohabitants of index smokers, could contribute to reducing COPD underdiagnosis. A recent systematic review and meta-analysis have shown that COPD underdiagnosis is a relevant problem, particularly when patients with respiratory symptoms and smoking exposure are considered. Likewise, the family setting may concentrate several factors associated with the risk of developing COPD. On the one hand, cohabitants of smokers are more likely to have been exposed to tobacco smoke and, in some cases, to have started smoking themselves^19^. On the other hand, exposure to environmental tobacco smoke may constitute a risk factor for developing this disease, even in people who have never smoked^6,30^.

Previous screening initiatives targeting unselected high-risk individuals or patients referred from specialized consultations have shown variable diagnostic yields^16,17^. The detection rate observed in our sample (24.4%) is in line with or exceeds the yields of opportunistic screening in the primary care setting (which usually range between 15% and 20%)^14,15,19,20^. However, these comparisons between studies should be interpreted with caution. Discrepancies between the diagnostic rates of our strategy and those reported in previous studies may be explained by heterogeneity in study design, inclusion criteria, and the screening tools used (spirometry versus portable microspirometers or risk questionnaires), as well as by differences in the characteristics of the study population.

As in previous studies, the implementation of this type of strategy in at-risk populations identified a high proportion of cases at early functional or clinical stages (58.5% at GOLD stage I and 62.2% with a low-risk profile)^17,20^. This allows for the early implementation of non-pharmacological interventions (physical activity, smoking cessation, education) and the early initiation of long-acting bronchodilator therapy (LABA or LAMA), which may modify the prognosis of COPD by preserving lung function and reducing healthcare resources in the long-term, including Emergency Department visits, hospitalizations, and pharmaceutical expenditure^31^.

Consistent with epidemiological studies, COPD patients in our cohort had a greater number of years of tobacco exposure^17^. Interestingly, we also identified a distinct subgroup of patients with pre-COPD. Although most had a history of smoking, one-quarter were non-smokers but had substantial passive tobacco exposure. Longitudinal follow-up of this sub-cohort will be relevant to characterize the risk of progression toward chronic airflow obstruction^28,29^.

Another relevant finding was the significant number of smoking cessation attempts observed among the cohabiting smokers included in this strategy. One possible explanation is that the information obtained through spirometry, including the estimation of lung age, may increase individual perception of the risk associated with smoking. In a randomized clinical trial conducted in 561 smokers, Parkes et al.^32^ observed that communicating lung age doubled the likelihood of smoking cessation. Other studies conducted in pulmonary function laboratories have shown similar results, although the motivational impact appears to depend, at least in part, on the values obtained and the difference between lung age and chronological age^33^. A lower frequency of smoking cessation attempts has been described in family environments where tobacco consumption is prevalent^34^. In this regard, addressing smoking cessation treatment across the entire family unit could strengthen interpersonal commitment and motivation, as well as facilitate the adoption of smoke-free policies, factors strongly associated with smoking cessation^35^.

Nevertheless, our study did not include a specific analysis that would allow us to determine whether the participation of cohabitants had a direct effect on smoking cessation among index smokers. Therefore, any potential influence of cohabitants should be considered a plausible hypothesis, rather than a causal relationship demonstrated by our results.

### Strengths and limitations

This study has several strengths. First, its multicenter design included different geographical areas within the same healthcare district, providing some heterogeneity to the study population. Second, the intervention was carried out in a real-world healthcare setting and involved the coordinated participation of primary care and specialized COPD and smoking cessation units. Third, the accessibility of primary care centers facilitated the recruitment of cohabitants, making it possible to extend the search for respiratory disease beyond the index smoker. Finally, the final number of participants exceeded the initial sample size estimate, providing more information than initially anticipated.

Nevertheless, several limitations should be considered. First, the opportunistic recruitment of smokers attending primary care and their cohabitants may have introduced selection bias and limits the generalizability of the results to the general population. Individuals who come into contact with the healthcare system may differ from those who do not in terms of age, smoking burden, presence of respiratory symptoms, risk perception, or motivation to quit smoking. However, this type of recruitment also reflects the conditions under which a strategy of this nature could be implemented in routine clinical practice and therefore provides relevant information regarding its feasibility in Primary care.

Second, the assessment of COPD was cross-sectional; therefore, it allows the identification of disease or pre-COPD at the time of assessment but does not allow causal relationships to be established between risk factors and diagnosis. This is particularly relevant for never smoker participants exposed to environmental tobacco smoke, in whom our results are compatible with a possible role of passive exposure but do not demonstrate causality.

Third, the single-arm design and absence of a comparator group limit the ability to determine the efficacy of the smoking cessation intervention. The cessation attempts and quit rates observed cannot be attributed exclusively to the intervention, as baseline motivation, spontaneous behavioral changes, or other contacts with healthcare professionals may have influenced the outcomes. For the same reason, it cannot be established that the participation of cohabitants had a direct effect on smoking cessation among index smokers.

Fourth, the six-month follow-up allows the assessment of initial and medium-term outcomes but does not allow sustained long-term abstinence or relapse rates to be determined. Nevertheless, this period provides clinically relevant information on the initial evolution of participants and allows the feasibility of follow-up within primary care to be assessed.

Finally, although the final sample size exceeded the initial estimate, the sample was relatively small, and not all eligible cohabitants from each family unit could be included. This limits the precision of the estimates and the possibility of conducting detailed subgroup analyses. Larger prospective studies, with more representative populations, comparator groups, and longer follow-up periods, will be necessary to confirm these results and determine the long-term effect of this type of strategy.

## Conclusions

The implementation of a multidisciplinary strategy integrating active COPD case finding and smoking cessation intervention among smokers and their cohabitants made it possible to identify new cases of this disease, predominantly at early stages, as well as a group of participants with pre-COPD. The identification of COPD in some cohabitants who had never smoked but had been exposed to environmental tobacco smoke highlights the potential relevance of considering the family environment when assessing the risk of developing this condition.

Among the cohabiting smokers included in the intervention, a greater number of smoking cessation attempts and quit attempts were also observed during follow-up. However, due to the single-arm design and the absence of a comparator group, these results should be interpreted as observed associations and do not allow us to establish that the intervention was the direct cause of changes in smoking behavior. Similarly, the present study does not allow us to determine whether the participation of cohabitants directly influenced smoking cessation among index smokers.

Overall, the results suggest that Primary care may constitute a suitable setting for integrating COPD detection and smoking cessation intervention, including cohabitants as a population potentially eligible for assessment. Nevertheless, the characteristics of the sample, opportunistic recruitment, the cross-sectional assessment of COPD, the absence of a control group, and the six-month follow-up limit the generalizability and causal interpretation of the results. Prospective studies with larger sample sizes, comparator groups, and longer follow-up are needed to confirm these findings and determine the long-term impact of this integrated approach.
