# Supplementary material for: Family based screening and smoking cessation for COPD detection: Hybrid cross-sectional and single-arm, multicenter intervention study
Source: Tob Induc Dis. 2026 Oct 22;24:10.18332/tid/235829. doi: 10.18332/tid/235829 (PMC13715596; doi:10.18332/tid/235829)
Supplement: Supplementary file 1 [file TID-24-196-s001.pdf]

Tobacco Induced Diseases

Supplementary file

© 2026 Rábade-Castedo C. et al.

DOI:
10.18332/tid/235829

The content has been provided by the author(s) and has not been reviewed, verified, or endorsed by European Publishing. It may not have undergone peer review. The views, opinions, and recommendations expressed are solely those of the author(s) and do not necessarily reflect the position of European Publishing. European Publishing accepts no responsibility or liability for any consequences arising from the use of, or reliance on, this content.

SUPPLEMENTARY FILE

Supplementary Table S1: STROBE Statement Checklist of items that should be included in reports of observational studies

Item	STROBE Section	Status	Observations & Manuscript Evaluation
**TITLE AND ABSTRACT**			
**1a**	Title	**Met**	Clearly says that it is an observational study, mentioning its design
**1b**	Abstract	**Met**	Clearly summarizes the objectives, multicenter setting, sample size (n=217), main findings (aOR and 95% CI).
**INTRODUCTION**			
**2**	Background / Rationale	**Met**	Solidly contextualizes COPD burden, underdiagnosis, and the rationale for screening exposed cohabitants within the household.
**3**	Objectives	**Met**	Clearly specifies the primary objective (COPD diagnosis via household screening and feasibility assessment).
**METHODS**			
**4**	Study design	**Met**	Defines the prospective, multicenter (8 primary care centers), cross-sectional, single-arm intervention design.
**5**	Setting	**Met**	Details the health area (Santiago-Barbanza), recruitment period (Sep 2023–Sep 2024), and 2-phase workflow.
**6**	Participants	**Met**	Clear inclusion/exclusion criteria for index cases and cohabitants.
**7**	Variables	**Met**	Thoroughly defines demographic, smoking, spirometric, clinical (mMRC, CAT, GesEPOC), and cessation variables.
**8**	Data sources / Measurement	**Met**	Describes standardized tools (CareFusion spirometer, co-oximetry, urinary cotinine, Fagerström test, etc.).
**9**	Bias	**Met**	Describes standardized tools (CareFusion spirometer, co-oximetry, urinary cotinine, Fagerström test, etc.).
**10**	Study size	**Met**	Justifies the calculated sample size (n=202, 3% precision, 95% CI) and adjustment to 238 to account for a 15% drop-out rate.
**11**	Quantitative variables	**Met**	Details the handling and categorization of continuous and ordinal variables (pack-years, scale scores).
**12**	Statistical methods	**Met**	Explains descriptive statistics, bivariable tests and multivariable logistic regression (aOR and 95% CI).
**RESULTS**			
**13**	Participants & Flow	**Met**	Reports complete participant flow (257 recruited → 40 excluded → 217 analyzed across 104 households).
**14**	Descriptive data	**Met**	Table 1 provides comprehensive sociodemographic, clinical, and smoking exposure characteristics for index cases and cohabitants.
**15**	Outcome data	**Met**	Reports COPD diagnosis rates (24.7% overall; 15% in cohabitants), GOLD/GesEPOC distribution, and smoking cessation rates.
**16**	Main results	**Met**	Presents univariable (Table 2) and multivariable (Table 3) analyses reporting adjusted ORs and 95% CIs for key predictors.
**17**	Other analyses	**Met**	Includes stratified subgroup analyses (cohabitants, active smokers vs. ex/non-smokers, Pre-COPD, PRISm).
**DISCUSSION**			
**18**	Key results	**Met**	Summarizes key findings at the beginning of the discussion
**19**	Limitations	**Met**	Transparently addresses study limitations (moderate sample size..
**20**	Interpretation	**Met**	Appropriately contextualizes primary care feasibility and motivational impact of lung age feedback compared to existing literature.
**21**	Generalizability	**Met**	Reflects on external validity of the multicenter approach and recommends replication in other health districts.
**OTHER INFORMATION**			
**22**	Funding & Ethics	**Met**	Ethics committee approval is explicitly cited (CEIC 2022/295). Adding a formal Funding/Conflicts of Interest heading is recommended.

Supplementary Table S2: COPD Screening Questionnaire (COPD-PS)

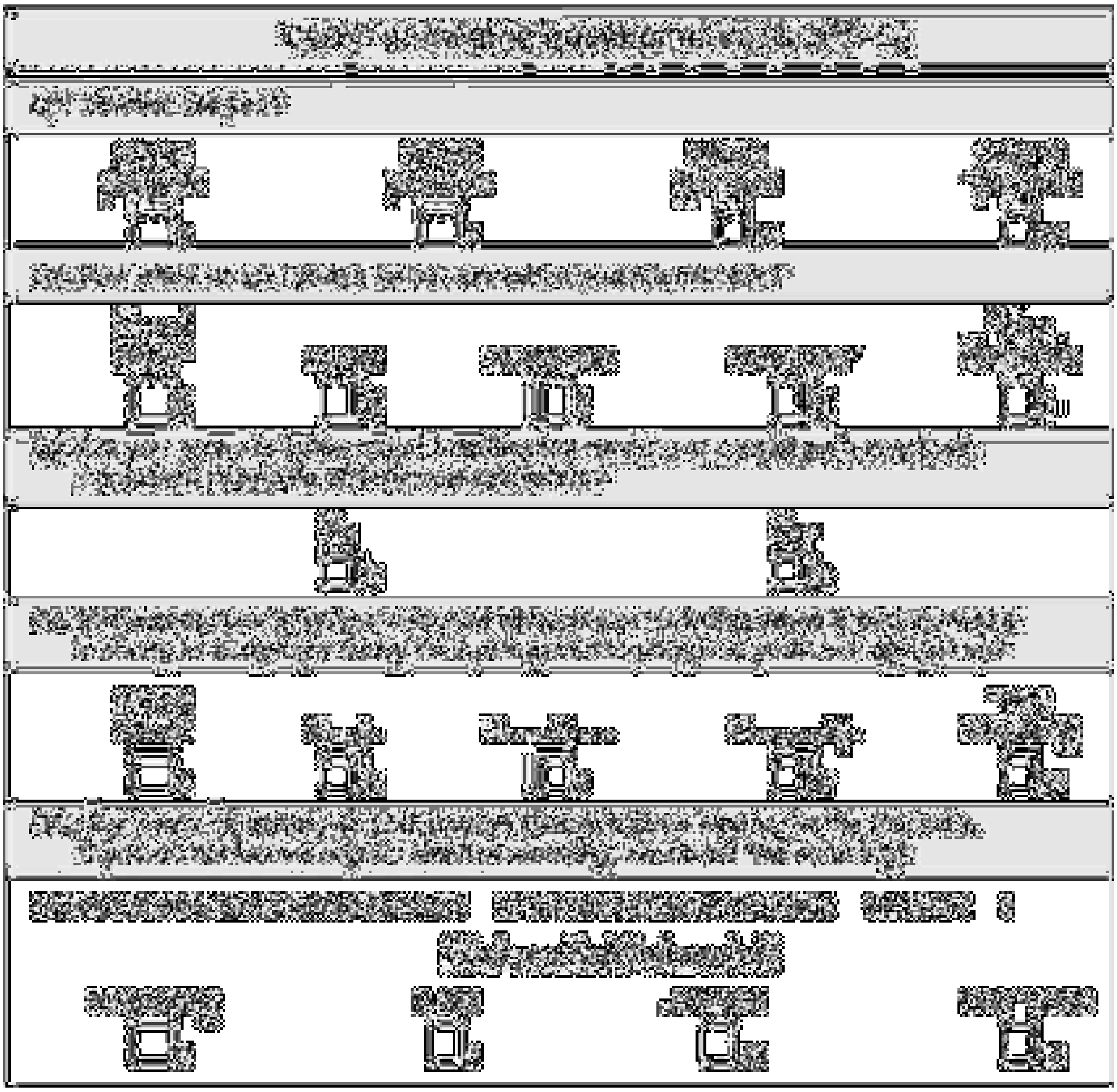

Supplementary Table S3: Family Smoke Exposure Questionnaire

1.	In the last 20 years, have you lived with smokers?
2.	Relationship
3.	Length of cohabitation
4.	Daily amount smoked by the cohabitant in your presence

Supplementary Table S4: Distribution and Definitions of Newly Diagnosed COPD Cases (N = 53) According to GesEPOC 2025 and GOLD Classifications

Values are expressed as frequency (n) with percentage (%) . Abbreviations: FEV1, Forced Expiratory Volume in 1 second

Classification / Category	Definition / Diagnostic Criteria	Patients (n)	Percentage (%)
**GesEPOC 2025 (Risk Phenotypes)**			
**Low-Risk**	Mild dyspnea (mMRC 0–1), FEV1 ≥ 50% predicted, and ≤ 1 moderate exacerbation/year (no hospitalization)	33	62.23%
**High-Risk Non-Exacerbator**	Meets high-risk criteria (FEV1 < 50% or mMRC ≥ 2) but has ≤ 1 moderate exacerbation/year (no hospitalization)	10	18.89%
**High-Risk Eosinophilic Exacerbator**	High-risk with ≥ 2 moderate exacerbations or ≥ 1 severe (hospitalized) exacerbation/year, and blood eosinophils ≥ 300 cells/µL	7	13.21%
**High-Risk Non-Eosinophilic Exacerbator**	High-risk with ≥ 2 moderate exacerbations or ≥ 1 severe (hospitalized) exacerbation/year, and blood eosinophils < 300 cells/µL	3	5.67%
**GOLD Spirometric Severity**			
**GOLD 1 (Mild)**	Post-bronchodilator FEV1 ≥ 80% predicted	31	58.5%
**GOLD 2 (Moderate)**	50% ≤ Post-bronchodilator FEV1 < 80% predicted	15	28.3%
**GOLD 3 (Severe)**	30% ≤ Post-bronchodilator FEV1 < 50% predicted	4	7.5%
**GOLD 4 (Very Severe)**	Post-bronchodilator FEV1 < 30% predicted	3	5.7%
**GOLD Clinical Groups (A, B, E)**			
**Group A**	Low symptom burden (mMRC 0–1 or CAT < 10) and low exacerbation risk (0 or 1 moderate exacerbation without hospitalization)	27	50.9%
**Group B**	High symptom burden (mMRC ≥ 2 or CAT ≥ 10) and low exacerbation risk (0 or 1 moderate exacerbation without hospitalization)	16	30.2%
**Group E**	High exacerbation risk (≥ 2 moderate exacerbations or ≥ 1 severe exacerbation requiring hospitalization), regardless of symptom score	10	18.9%
